# Gene co-expression analysis unravels a link between *C9orf72* and RNA metabolism in myeloid cells

**DOI:** 10.1186/s40478-015-0242-y

**Published:** 2015-10-15

**Authors:** Serge Nataf, Laurent Pays

**Affiliations:** Lyon 1 University, CarMeN Laboratory, INSERM U-1060, INRA USC-1235, 69921 Oullins, France; Banque de Tissus et de Cellules des Hospices Civils de Lyon, Hôpital Edouard Herriot, Place d’Arsonval, 69003 Lyon, France

GGGGCC hexanucleotide repeat expansion in the promoter or intronic regions of *C9orf72* is responsible for the most common familial forms of amyotrophic lateral sclerosis (ALS) and frontotemporal lobar degeneration (FTLD) [4]. Gain-of-function of *C9orf72*, at the mRNA and/or protein level, is currently considered as a major mechanism of neurodegeneration in these patients [2, 5, 7]. To further elucidate the genomic impact of a *C9orf72* gain-of-function, we performed a gene co-expression analysis using the open source bioinformatics tool Multi Experiment Matrix (MEM) [1] that covers a large set of human transcriptomic data (*n* = 1794) on the same expression array platform (Affymetrix HG-U133_Plus_2). This approach allowed us to identify the 100 mRNA species that are overall the most positively correlated with *C9orf72* mRNA levels and, conversely, the 100 mRNA species that are the most inversely correlated with *C9orf72* mRNA levels. We then used “EnrichR” [3] to assess these two gene lists with regard to their enrichment in subsets of genes sharing the same Gene Ontology (GO) annotations i.e. belonging to the same functional family. While we did not find any significant enrichment in the list of genes whose expression levels were positively correlated with *C9orf72*, the list of mRNA species that were inversely correlated with *C9orf72* was highly significantly enriched in genes annotated with RNA metabolism-related GO terms. These included notably the terms “ncRNA metabolism” (adjusted *p-value* = 6.57E-6), “tRNA aminoacylation” (adjusted *p-value* = 6.57E-6) and “tRNA metabolic process” (1.90E-5). Table 1 shows the full list of GO terms for which a significant enrichment with an adjusted *p*-value < 0.001 was found. This data shows that an increase of *C9orf72* mRNA levels associates with a concomitant downregulation of genes that exert key functions in RNA metabolism. Altered RNA metabolism is considered as a key pathological feature in not only *C9orf72* mutation carriers but also patients bearing mutations in *FUS* or *TDP43* genes as well as sporadic ALS patients [8]. Our observation suggests that an increased expression of non-mutated *C9orf72* may similarly trigger RNA metabolism alterations. However, the relevance of such a finding in the context of *C9orf72* mutation remains to be determined.

**Table 1:** Enrichment analysis of genes inversely correlated with *C9orf72* mRNA levels

GO Term	Adjusted *p*-value	Genes
ncRNA metabolic process (GO:0034660)	6.57E-06	*TRUB2;POP1;PARS2;PA2G4;EPRS;PDCD11;GEMIN5;TARS2;UTP20;IARS;FARSA;EARS2;AARS*
tRNA aminoacylation for protein translation (GO:0006418)	6.57E-06	*PARS2;TARS2;IARS;EPRS;FARSA;EARS2;AARS*
tRNA aminoacylation (GO:0043039)	6.57E-06	*PARS2;TARS2;IARS;EPRS;FARSA;EARS2;AARS*
amino acid activation (GO:0043038)	6.57E-06	*PARS2;TARS2;IARS;EPRS;FARSA;EARS2;AARS*
cellular amino acid metabolic process (GO:0006520)	1.04E-05	*PYCRL;PARS2;CAD;PYCR2;CTPS1;EPRS;PFAS;GLS;TARS2;IARS;FARSA;GART;EARS2;AARS*
tRNA metabolic process (GO:0006399)	1.90E-05	*TRUB2; POP1;PARS2;TARS2;IARS;EPRS;FARSA;EARS2;AARS*
glutamine family amino acid metabolic process (GO:0009064)	0.0002	*PYCRL;CAD;PYCR2;CTPS1;PFAS;GLS*

A gene enrichment analysis was performed on the list of top 100 genes that are the most inversely correlated with *C9orf72* mRNA levels. Left column: GO terms for which enrichment was found; middle column: *p*-values adjusted from Fisher exact test; right column: *C9orf72* inversely correlated genes annotated with the corresponding GO term. *AARS*: alanyl-tRNA synthetase; *CAD*: carbamoyl-phosphate synthetase 2, aspartate transcarbamylase, and dihydroorotase; *CPTS1*: CTP synthase 1; *EARS2*: glutamyl-tRNA synthetase 2, mitochondrial; *EPRS*: glutamyl-prolyl-tRNA synthetase; *FARSA*: phenylalanyl-tRNA synthetase, alpha subunit;* GART*: phosphoribosylglycinamide formyltransferase, phosphoribosylglycinamide synthetase, phosphoribosylaminoimidazole synthetase; *GEMIN5*: gem (nuclear organelle) associated protein 5; *GLS*: glutaminase; *IARS*: isoleucyl-tRNA synthetase; *PA2G4*: proliferation-associated 2G4, 38kDa; *PARS2*: prolyl-tRNA synthetase 2, mitochondrial (putative); *PDCD11*: programmed cell death 11;* PFAS*: phosphoribosylformylglycinamidine synthase; *POP1*: processing of precursor 1, ribonuclease P/MRP subunit (S. cerevisiae);* PYCR2*: pyrroline-5-carboxylate reductase family, member 2; *PYCRL*: pyrroline-5-carboxylate reductase-like; *TARS2*; threonyl-tRNA synthetase 2, mitochondrial (putative); *TRUB2*: TruB pseudouridine (psi) synthase family member 2; *UTP20*: UTP20, small subunit (SSU) processome component, homolog (yeast)

Interestingly, among the 1794 microarray expression studies from which *C9orf72* inverse correlations were calculated, data sets analyzing the transcriptomic profile of myeloid cells, in particular acute myeloid leukemia cells, were by far the most informative i.e. giving rise to the most significant inverse correlations. In addition, it is worth noting that in the BioGPS Affymetrix expression atlas [9], *C9orf72* probes are reported to detect much higher *C9orf72* mRNA levels in monocytes than in neurons or astrocytes. Monocytes belong to the myeloid lineage and share many phenotypic and functional properties with microglia, although both cell types derive from distinct progenitors [6]. Therefore, one may consider that a link between *C9orf72* and RNA metabolism could similarly occur in microglia. This deserves further investigation. Finally, our observation suggests that *C9orf72* is possibly a key regulator of RNA metabolism in acute myeloid leukemia cells.
